# Robot-Assisted Nephroureterectomy for Upper Tract Urothelial Carcinoma in a Patient with an Ileal Conduit

**DOI:** 10.1155/2022/5321613

**Published:** 2022-05-04

**Authors:** Raymond A. Stemrich, Neel Hasmukh Patel, Jacob A. Baber, Mark J. Ferretti

**Affiliations:** ^1^Geisinger Health System, 100 N. Academy Avenue, Danville, PA 17822, USA; ^2^Geisinger, Urology Associate, 1000 E. Mountain Boulevard, Wilkes-Barre, PA 18702, USA

## Abstract

**Background:**

Upper tract urothelial carcinoma remains an uncommon disease that is clinically difficult to identify early and surveil. Open nephroureterectomy is the gold standard for patients with high-grade disease, especially for patients in whom surveillance is complicated such as those with prior cystectomies/ileal conduits. This report presents a case of a patient with a history of radical cystectomy and ileal conduit construction who underwent a successful minimally invasive robotic surgery for treatment of upper tract urothelial carcinoma. *Case Presentation*. The patient is a 72-year-old Caucasian male with a history of recurrent superficial bladder tumors treated with cystoscopies with fulguration, Bacillus Calmette-Guerin, and a robot-assisted cystectomy with ileal conduit diversion presenting with recurrent urinary tract infections and hematuria secondary to a ureteral stricture. The patient was admitted previously for urosepsis during which time a percutaneous nephrostomy tube was inserted on the right side. Upon presentation, imaging revealed a lesion extending from the lower pole of the right kidney into the renal pelvis. The presence of a nephrostomy and urostomy allowed the surgical team to utilize a minimally invasive approach to remove the diseased kidney and ureter with visualization enhanced by indocyanine green.

**Conclusion:**

Minimally invasive robot-assisted approaches to treating upper tract urothelial carcinomas may offer an alternative to the open cases typically employed in cases of patients with prior ileal conduit. Furthermore, utilizing indocyanine green may expand the applicability of such approaches to uro-oncologic cases with greater complexity.

## 1. Introduction

Upper tract urothelial carcinoma (UTUC) remains an uncommon condition making up only 5% of urothelial carcinomas. Radical nephroureterectomy, whether an open, laparoscopic, or robotic approach, remains the standard treatment [1]. Although some aspects of these modalities demonstrate comparable perioperative, postoperative, and oncologic outcomes, further consideration is required before choosing an approach.

The laparoscopic and robotic approaches are minimally invasive with inherent advantages over open procedures, particularly in perioperative outcomes. Previous studies have found that minimally invasive nephroureterectomies require less intraoperative time and result in less mean blood loss. Postoperatively, they require shorter hospital stays, produce better cosmetic outcomes, and require less postoperative analgesics. Postoperative complications and oncologic outcomes were found to be comparable between the approaches [2, 3].

When comparing minimally invasive modalities, the robotic approach has been found to have longer operative times thought to be caused by the initial docking process [2, 4]. Furthermore, Veccia et al. found that the robotic approach had lower overall complications and shorter recovery times; however, these results were not statistically significant [4]. Other studies comparing oncologic outcomes (i.e., progression-free, cancer-specific, and overall survival) concluded that laparoscopic and robotic approaches were comparable [2, 4, 5]. The main advantages of the robotic approach include increased dexterity, improved accuracy, and enhanced visualization intraoperatively [2, 4]. Veccia et al. defined their “tetrafecta” outcome for robotic nephroureterectomies as concomitant occurrence of bladder cuff excision, lymph node dissection, no complications, and negative surgical margins as their measure of quality. They concluded that the improved versatility of robotic platforms overcomes the challenges with laparoscopic approaches achieving their “tetrafecta” [4].

This report presents the case of a patient who underwent a cystectomy with ileal conduit creation who subsequently developed UTUC. The surgical team elected to perform a robot-assisted nephroureterectomy with the use of indocyanine green (ICG) to improve visualization of the ureter and ileal conduit.

## 2. Case Presentation

**Figure 1:**
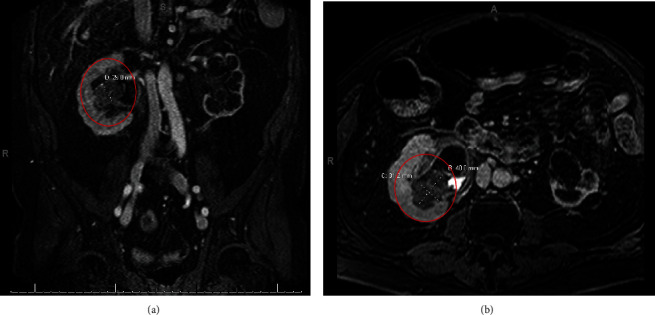
(a) MR Urogram showing the coronal view of the lesion measuring 0.29 mm. (b) MR Urogram showing the axial view of the lesion measuring 31.2 mm × 40.0 mm.

A 72-year-old Caucasian male presented with intermittent hematuria and recurrent urinary tract infections secondary to a right ureteral stricture. The patient's significant past medical history included type 2 diabetes mellitus, dyslipidemia, coronary artery disease, stage 5 chronic kidney disease, and bladder cancer. He was initially diagnosed with superficial bladder carcinoma in 2000 and underwent multiple cystoscopies with fulguration of bladder tumors and intravesical Bacillus-Calmette-Guerin before a robot-assisted cystoprostatectomy was performed in 2010. An ileal loop diversion was performed with a Bricker ureteroileal anastomosis. Additionally, obturator and iliac lymph node dissections were performed, but no involvement was observed. Pathology at the time revealed extensive urothelial atypia consistent with high-grade carcinoma in situ. There was no evidence of an invasive process, but the right ureteral margin was positive for low-grade dysplasia. The final pathology report classified it as stage tis. After admission for urosepsis and placement of a right percutaneous nephrostomy (PCN), the patient presented to our clinic. MR Urogram demonstrated a right ureteroileal anastomosis stricture and a lobulated mass in the right kidney extending from the lower pole into the renal pelvis (Figure 1). Biopsy demonstrated high-grade urothelial carcinoma.

Preoperatively, a CT scan revealed that the patient's left kidney was severely atrophic and nonfunctioning. The planned removal of the right kidney would require the patient to undergo dialysis. Surgical options were discussed with the patient, including the possibility of a kidney-sparing procedure with aggressive endourologic resection and instillation of MitoGel. Ultimately, this was decided against due to the patient's worsening symptomatic azotemia. Upon presentation to the clinic, the patient's GFR was 14.2 and his creatinine was 3.8 mg/dL. Additionally, he was experiencing hypertension (systolic 152-178 mmHg, diastolic 72-84 mmHg) and hyperkalemia. On preoperative evaluation, his GFR declined to 8.5 and his creatinine was 4.5 mg/dL (renal function values in Table 1). In consultation with the patient's nephrologist, it was determined that dialysis was inevitable with his progressive renal impairment, so the patient elected to undergo a nephroureterectomy. A right-sided PCN and right lower quadrant urostomy allowed the surgical team to plan a robot-assisted nephroureterectomy utilizing indocyanine green (ICG) to improve visualization of the ureter and ileal conduit.

**Figure 2:**
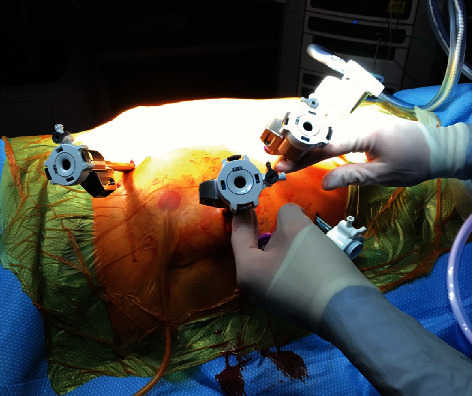
Placement of the ports.

**Figure 3:**
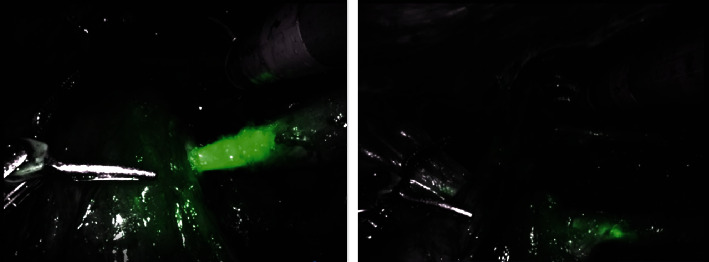
View of the ureter entering the ileal conduit after injection of indocyanine green (ICG).

The patient was placed in a modified flank position and flexed at the hip. An 18F Foley catheter was inserted into the ileal conduit before the abdomen was insufflated and a Visiport, three robotic trocars, and two assistant trocars were inserted (Figure 2). After docking the da Vinci Xi® platform, a standard approach was employed until both poles of the kidney were visible at which time ICG was injected into the nephrostomy to improve visibility of the proximal ureter (Figure 3). At this point, the ureter was clipped, and the nephrectomy continued. Once the ureter was dissected to the level of the iliac vessels, ICG was injected through the urostomy to confirm the location of the ileal conduit and allow visualization of the anastomoses. A clip was placed on the distal ureter prior to entry into the conduit and excision of the conduit cuff around the ureter. The case was concluded in the usual fashion with the right kidney, ureter, and conduit cuff inserted into an EndoCatch bag and removed from the body cavity. Pathology revealed an invasive high-grade papillary urothelial carcinoma infiltrating the lamina propria with a final staging of pT1pNX.

Overall, operative time was four hours. Perioperative findings included moderate peritoneal adhesions and estimated blood loss of 50 mL with grossly negative margins. The patient tolerated the procedure well, but he developed an ileus postoperatively, which prolonged his hospital stay. He was discharged on postop day 10 and did well in follow up. In addition to monitoring the patient's chronic anemia secondary to chronic kidney disease, recurrence is surveilled with urine cytology obtained from the ileal conduit and the left kidney.

## 3. Discussion

Radical cystectomy serves as the standard treatment for muscle invasive bladder cancer, requiring urinary diversion such as an ileal conduit. This approach is generally curative for bladder cancers; however, patients may develop UTUC with an overall postcystectomy prevalence of 0.75-6.4% [6]. This prevalence after radical cystectomies may represent an underestimate given the pan-urothelial field defect theory and the high mortality associated with the initial bladder cancer diagnosis [6, 7]. Regardless, regular monitoring of the patient postoperatively is essential to identify recurrence early.

Surveillance itself poses challenges and remains a topic of ongoing discussion within the field. One study assessed the use of cytology and various imaging techniques (i.e., loopgraphy, urography, and computerized tomography) in the detection of UTUC. The rate of primary detection with urine cytology was only 8.9%; furthermore, the results of analysis may be difficult to interpret because the sample is often obtained from an ileal conduit or another urinary diversion. Despite these potential ambiguities, urine cytology is an inexpensive test to monitor recurrence and remains widely used by many providers. The primary detection rate for imaging modalities was 24.8%; however, roughly eight hundred radiologic assessments were performed to identify one case of UTUC. Additionally, many of these tests are conducted in tandem with other imaging techniques, making assessing the specificity and sensitivity of the individual methods challenging [6]. Regardless of detection method, UTUC is primarily treated with surgery unless deemed inoperable.

The gold standard treatment for high-grade UTUC, especially for patients with a history of cystectomy, is a radical open nephroureterectomy with excision of the bladder cuff; however, minimally invasive approaches are becoming more common and yielding improved outcomes. Despite acceptance of robot-assisted procedures in the treatment of prostate, kidney, and bladder cancers, its oncological results for UTUC remain unmeasured in the long term and thus the approach is not widely validated. In a retrospective study, De Groote et al. concluded that the minimally invasive robotic approach was safe with a low rate of major complications and low perioperative morbidity. Furthermore, the robot-assisted method produced oncologic outcomes similar to those of laparoscopic and open nephroureterectomies in the short term [1]. Several factors need to be considered in the planning stage of any robotic surgery. In the era of immunotherapy and the availability of novel agents such as Jelmyto, upper tract disease is increasingly able to be managed with renal preservation. In patients with salvageable kidneys, this may become the preferred route for both high-grade and low-grade disease when feasible. With dialysis already being planned for this patient, our decision was made to proceed with nephroureterectomy after discussion with patient.

In the case described above, the surgical plan took advantage of the ileal conduit urostomy and PCN to inject ICG to enhance visibility of key structures. ICG has a wide range of uses. Most notably in urology, it is employed to visualize lymph nodes during dissection, perfusion in transplants, and anatomy in ureteral procedures [8]. The operative plan for the patient in this case, included injecting ICG into the nephrostomy tube to highlight the ureter proximally, enabling clip placement near the renal pelvis. It was then injected into the urostomy, allowing visualization of the conduit and ureteroileal anastomoses. This step improved clip placement on the distal ureter and excision of the conduit cuff. Overall, utilization of ICG augments visibility during robot-assisted cases expanding both the approach's applicability and safety.

## 4. Conclusion

We reported a patient with upper tract urothelial carcinoma who underwent a robot-assisted nephroureterectomy utilizing ICG via a nephrostomy and urostomy to improve visualization of the ureter and ileal conduit anastomoses. This case report contributes to the growing body of evidence supporting the efficiency, safety, and flexibility of minimally invasive robotic approaches in complex urologic cases.

## Figures and Tables

**Table 1:** Baseline renal function upon presentation to the clinic and renal function at the time of preoperative evaluation.

	Presentation to clinic	Preoperative evaluation
BUN (mg/dL)	72.0	36.0
Creatinine (mg/dL)	3.8	4.5
Estimated GFR	15.0	12.5
